# A Comparative Analysis of Metabolic Profiles of Embryonic Skeletal Muscle from Lantang and Landrace Pigs

**DOI:** 10.3390/ani12040420

**Published:** 2022-02-10

**Authors:** Shufang Cai, Tianqi Duo, Xiaoyu Wang, Xian Tong, Chenglong Luo, Yaosheng Chen, Jianhao Li, Delin Mo

**Affiliations:** 1 State Key Laboratory of Livestock and Poultry Breeding & Guangdong Public Laboratory of Animal Breeding and Nutrition & Guangdong Key Laboratory of Animal Breeding and Nutrition, Institute of Animal Science, Guangdong Academy of Agricultural Sciences, Guangzhou 510640, China; csfsfc0401@163.com (S.C.); luochenglong@gdaas.cn (C.L.); 2 State Key Laboratory of Biocontrol, School of Life Sciences, Sun Yat-Sen University, Guangzhou 510006, China; duotianqi@163.com (T.D.); wangxy067@163.com (X.W.); tongxian_jiangsu@163.com (X.T.); chyaosh@mail.sysu.edu.cn (Y.C.)

**Keywords:** pig, GC–MS, skeletal muscle, TCA cycle, nucleotide metabolism, energy metabolism

## Abstract

**Simple Summary:**

The pig is one of the most important domesticated meat animals. Some studies have revealed that pigs with low meat production show more intense myogenesis at the early stage of embryonic muscle development than pigs with high meat production. Here, by gas chromatography–mass spectrometry GC–MS based metabolomics, we concluded that the nucleotide metabolism and energy metabolism of the *longissimus* *lumborum* (*LL*) were increased in Lantang pigs compared with Landrace pigs, indicating rapid synthesis of nucleic acids and ATP to meet the material and energy requirements of rapid cell proliferation and differentiation in Lantang pigs.

**Abstract:**

Elucidation of the complex regulation of porcine muscle development is key to increasing pork output and improving pork quality. However, the molecular mechanisms involved in early porcine embryonic muscle development in different pig breeds remain largely unknown. Here, GC–MS based metabolomics and metabolomic profiling was used to examine the *longissimus lumborum* (*LL*) of the Lantang (LT) and the Landrace (LR) pig at embryonic day 35 (E35). Metabolites showed clear separation between LT and LR, with 40 metabolites having higher abundances in LT and 14 metabolites having lower abundances in LT compared with LR. In addition, these metabolic changes were mainly associated with nucleotide metabolism and energy metabolism, such as purine metabolism, pyrimidine metabolism, the pentose phosphate pathway, and the TCA cycle. More interestingly, the contents of DNA, RNA, and ATP per unit mass of *LL* tissues were higher in LT, indicating rapid synthesis of nucleic acids and ATP, to meet both the material and energy requirements of rapid cell proliferation and differentiation. Furthermore, enzyme activity associated with the TCA cycle and pentose phosphate pathway, including α-ketoglutaric dehydrogenase (KGDH), malate dehydrogenase (MDH), pyruvate dehydrogenase (PDH), succinate dehydrogenase (SDH), and glucose-6-phosphate dehydrogenase (G6PDH), were higher in LT. Based on these results, we conclude that there are significant differences in nucleotide metabolism and energy metabolism of *LL* between LT and LR, and we speculate that the enhanced nucleic acid metabolism and energy metabolism in LT can meet the material and energy requirements of rapid cell proliferation and differentiation, making myogenesis more intense in LT compared to LR which might be the metabolic mechanism underlying the distinct skeletal muscle development in the two breeds.

## 1. Introduction

The pig is an important domesticated meat animal and it is also a useful model for numerous human muscular diseases, therefore, a more thorough understanding of porcine skeletal muscle development is informative [1,2]. Myogenesis is a highly complex biological process that can be divided into three periods: primary myogenesis from E35 to E64, secondary myogenesis from E54 to E90 [3], and a third generation of fibers around birth [4,5]. Differences in muscle development between pig breeds are associated with their specific qualitative and quantitative traits quality and quantity traits [6,7]. Analysis of global gene expression profiles has identified genes related to porcine meat quality and muscle growth [8,9,10]. It is also known that miRNAs and their muscle-specific targets play a significant role in myogenic differentiation [11,12,13]. In addition, any alteration in intracellular signaling pathways may result in muscular diseases, such as skeletal muscle atrophy and pathological hypertrophy [14,15,16]. Thus, clarification of the complex regulation of porcine muscle development is valuable in the improvement of meat quality and production, and it might also contribute to the prevention and treatment of human muscle diseases.

Lantang (LT) is an indigenous lard-type pig breed grown in southern China, which is characterized by delicious meat, high intramuscular fat content, and early sexual maturity. Landrace (LR) is a commercial meat-type pig breed with high lean meat percentage, fast muscle growth and high body weight [17]. Because of the large differences in muscle development between these two breeds, LT and LR are useful models to study the mechanisms of muscle development between different breeds [18,19,20,21]. In our previous study regarding the contrast in muscle development between LT and LR, the results revealed that primary muscle fibers appear earlier in LT than in LR at E35 and E42, respectively [22]. Recent reports indicated that the process of muscle formation is more intense in Meishan pigs (Chinese indigenous miniature pig) than that in Large White pigs (a European lean breed) at E35 [23]. These studies revealed that pigs with lower levels of meat production showed more intense myogenesis at the early stage of embryonic muscle development than pigs with high meat production, but the mechanisms concerning this phenomenon have not been well elucidated.

Metabolomics has emerged as a powerful tool for exploring metabolic processes, identifying crucial metabolic biomarkers, and revealing metabolic mechanisms during cell growth, development, and senescence [24,25]. Furthermore, metabolic characterization of a well-defined group of patients is beneficial for revealing metabolic signatures to explain muscle weakness in chronic diseases [26]. In addition, some studies have identified metabolites associated with lean mass or body mass index (BMI) [27,28,29]. The association between metabolites and muscle mass in a healthy, elderly Taiwanese population has been explored. The results showed that the higher catabolic rate of amino acids was linked with muscle-mass loss [30]. Studies have revealed that the characteristics of the skeletal muscle metabolome, neuromuscular disease, and daily changes in tissue metabolites during strenuous exercise in humans are associated with daily nutrient levels and aging [31,32]. Metabolic gene signatures associated with different myogenic cell cycle states were also associated with metabolic signatures of mitochondrial function in young and aged mouse muscle satellite cells [33,34]. These studies suggest that metabolic requirements are critically linked to myogenic cell fates and skeletal muscle homeostasis. However, few metabolomics studies have addressed porcine embryonic muscle development.

In the present study, to gain further insight into the mechanism underlying the greater myogenic capacity in LT than in LR at the early embryonic stage, GC–MS based metabolomics was used to analyze metabolic profiles in *LL* of LT and LR in E35 embryos. Our study aimed to uncover the metabolic mechanisms associated with differences in early embryonic muscle development between LT and LR breeds, which could contribute to the improvement of pork output as well as the treatment and prevention of muscle diseases in humans.

## 2. Materials and Methods

### 2.1. Experimental Animals and Tissues

Six purebred LT sows with the same genetic background were inseminated with semen from purebred LT boars, and six purebred LR sows with the same genetic background were artificially inseminated with semen from purebred LR boars. All pigs were allowed ad libitum access to feed and water and were housed under identical conditions. All sows were slaughtered at 35 days after insemination, and all embryos of each sow were collected. There was no visual difference between the embryos or *LL* tissues of LT and LR at E35. For each sow’s embryo, the *LL* tissues were dissected. *LL* tissues from the same sow were cut up and mixed as the experimental samples. These samples were immediately snap-frozen in liquid nitrogen and stored until further use. For GC–MS detection, six biological replicates were used in the LT group and the LR group, each from a sow; for measurement of ATP, NADPH, and enzyme activity, at least three biological replicates were used in the LT group and LR group, each from one sow.

### 2.2. Metabolite Extraction and Derivatization

For metabolite extraction, we referred to a reported study [35]. In brief, tissue samples were stored at −80 °C and fully ground with a mortar and pestle in liquid nitrogen, and then transferred into 1.5 mL centrifuge tubes. Subsequently, 10 µL of ribitol (0.1 mg per mL) was added into each tube as an internal quantitative standard. From 100 mg samples, metabolites were extracted using 1000 µL cold methanol (100%, Sigma-Aldrich, St. Louis, MO, USA). The mixed samples were vortexed for 1 min, followed by centrifugation for 10 min at 12,000× *g*. After centrifugation, supernatants were transferred to a new Eppendorf tube and immediately concentrated in a rotary vacuum centrifuge device. For metabolite derivatization, the residue was dissolved in 100 µL methoxyamine pyridine solution (20 mg per mL) and incubated at 37 °C for 120 min in an incubator shaker. Lastly, each mixture sample was treated with 100 µL MSTFA reagent (containing 1% TMCS) and incubated at 37 °C for 30 min. All experiments were repeated using six biological replicates.

### 2.3. GC–MS Detection

GC–MS detection was performed with a variation on the two-stage technique [36]. By splitless injection, a 1μL derivatized sample was injected into a 30 m × 250 μm × i.d. 0.25 μm PH-5MS column (Agilent Technologies, Santa Clara, CA, USA). Then, analyses were conducted using an Agilent 7890A GC equipped with an Agilent 5975C VL MSD detector (Agilent Technologies, Santa Clara, CA, USA). The gas chromatography (GC) oven was set to an initial temperature of 85 °C for 5 min, then increased 15 °C per min for 5 min to 280 °C, followed by an increase to 310 °C at a rate of 20 °C per min. Helium was selected as the carrier gas, and the flow was maintained at 1 mL per min. MS was conducted in a range of 50–600 m/z.

### 2.4. Spectra Processing for GC/MS

After mass spectra were acquired, deconvolution and calibration were conducted by AMDIS (Agilent OpenLAB CDS ChemiStation C.01.01). Peaks with a signal-to-noise ratio (S/N) < 30 were excluded to avoid false positives [37]. The retention time was corrected, and compound peaks were aligned using the alkane standards (C11, C12, C13, C14, C15, C16, C17, C18, C19, C20, C22, C24, C26, C28, C30, C32, C34, and C36). Subsequently, artifact peaks were removed by comparing with the blank samples. Metabolites from the GC–MS spectra were identified by retrieval from the NIST 2011 (National Institute of Standards and Technology, Gaithersburg, MD, USA) library according to the National Institute of Standards. Metabolite abundance was calculated by taking the relative peak area value of adonitol as the internal standard [38]. The acquired data array file was used for further multivariate statistical analysis.

### 2.5. Bioinformatics Analyses

Data transformations and manipulations were performed using Excel, and included two core steps. The first was to convert the abundance of each metabolite from original data of GC–MS detection into an Excel table for subsequent analysis. Second, in the process of data standardization, the data matrix in Excel was calculated, which included the Log transformation, scaling, centraling, and other transformation involved in PCA and OPLS-DA analysis. Differential metabolites were obtained by comparing the two groups using the Mann–Whitney U test (*α* = 0.05) with SPSS 23.0 (IBM, Chicago, Illinois, USA). *Z*-value based on the LR was calculated to identify the differential abundance of metabolites of LT compared to LR. The *Z*-score plot spanned from −10.94 to 24,536.17 in LT. On the Metabo Analyst online website [39,40,41], principal component analysis (PCA) and orthogonal partial least squares-discriminant analysis (OPLS-DA) of the metabolomic data was performed. For *Z*-score analysis, each metabolite was scaled according to a reference distribution, and a calculation was conducted with the mean and standard deviation of reference sets as a control. Using the distance matrix, a hierarchical cluster analysis (HCA) was performed. Then, the relationships among the samples were tested by PCA and OPLS-DA analyses. Comparative metabolic pathway analysis between LT and LR groups was performed using the Metabo Analyst online website (www.metaboanalyst.ca/) (accessed on 20 February 2021). A hypergeometric test was used to calculate the −log(*p*) value for reflecting the impact of each metabolic pathway, and pathways with *p* < 0.05 were retained. Finally, data were represented by histogram and scatter plots using Prism v5.01 (GraphPad, La Jolla, CA, USA). Comparative metabolic pathway analysis between LT and LR was performed using iPath2.0 [42].

### 2.6. Measurement of Activity of α-Ketoglutaric Dehydrogenase (KGDH), Pyruvate Dehydrogenase (PDH), Malate Dehydrogenase (MDH), and Succinate Dehydrogenase (SDH)

The samples were fully ground in liquid nitrogen, and a 100 mg sample homogenized with 1 mL PBS (pH 7.4) was broken down by sonication (at a 200 W power) for 10 min on ice. The samples were then centrifuged at 12,000 rpm for 10 min to remove insoluble material. The protein concentration of the supernatants was measured by BCA kit (Beyotime Biotechnology, Shanghai, China), then an equivalent amount of proteins in the supernatants was vacuum freeze dried. The vacuum freeze dried powder containing 100 μg proteins were redissolved and transferred to a ketoglutarate dehydrogenase (KGDH) reaction mix (1 mM MgCl_2_, 0.5 mM MTT, 6.5 mM PMS, 50 mM alpha-ketoglutaric acid potassium salt, 0.2 mM TPP, and 50 mM PBS), pyruvate dehydrogenase (PDH) reaction mix (1 mM MgCl_2_, 0.5 mM MTT, 6.5 mM PMS, 2 mM sodium pyruvate, 0.2 mM TPP, and 50 mM PBS), succinate dehydrogenase (SDH) reaction mix (0.5 mM MTT, 5 mM sodium succinate, 13 mM PMS, and 50 mM PBS), and malate dehydrogenase (MDH) reaction mix (0.5 mM MTT, 5 mM sodium malate, 13 mM PMS, and 50 mM PBS) to a final volume of 200 µL in a 96-well plate. After incubation at 37 °C for 60 min, each plate was measured at 566 nm for absorbance. 

### 2.7. ATP Measurement

ATP content was quantified using an ATP Assay Kit (Beyotime, Shanghai, China). In brief, samples were lysed followed by sonication (750 W, operating at 40% power, 3 cycles of 5 s on and 5 s off), then centrifuged at 12,000× *g* for 10 min. The supernatants were used to measure the ATP level according to the manufacturer’s instructions. The produced luminescence was analyzed (Mithras, Mikrowin 2000 software, Berthold Technologies, Thoiry, France). Finally, the luminescence value was plugged into the standard curve to determine the ATP content.

### 2.8. Measurement of Nicotinamide Adenine Nucleotide Phosphate (NADPH)

NADPH levels were determined using a NADP+/NADPH Detection Kit (Beyotime, Shanghai, China). Tissue samples of 10 to 30 mg were homogenized on ice with 400 μL NADP+/NADPH-extracting solution, then centrifuged at 12,000× *g* for 10 min at 4 °C and 50 μL supernatants were used to measure NADPH. After being heated in a water bath at 60 °C for 30 min, each supernatant sample was added into 100 μL G6PDH working liquid in a 96-well plate and incubated at 37 °C for 10 min. Following this, color reaction was performed for 30 min. Finally, each plate was measured at 450 nm for the absorbance. 

### 2.9. Measurement of Activity of Glucose-6-Phosphate Dehydrogenase (G6PDH)

G6PDH activity was detected using a G6PDH activity assay kit (Beyotime, Shanghai, China) according to the manufacturer’s instructions. The reduction of NADP was measured spectrophotometrically at 450 nm.

### 2.10. Statistical Analysis

Measurement of ATP, NADPH, and enzyme activity was repeated at least three times independently, and representative results are shown. SPSS 23.0 (IBM, Chicago, IL, USA) and Prism v5.01 (GraphPad, La Jolla, CA, USA) were utilized for statistical analyses. Differences between groups were assessed for significance with the *t*-test, and the results are presented as mean ± SEM. Asterisks (*) indicate levels of statistical significance. * *p* < 0.05; ** *p* < 0.01; *** *p* < 0.001.

## 3. Results

### 3.1. Metabolomic Profiling of LL in LT and LR at E35

**Figure 1:**
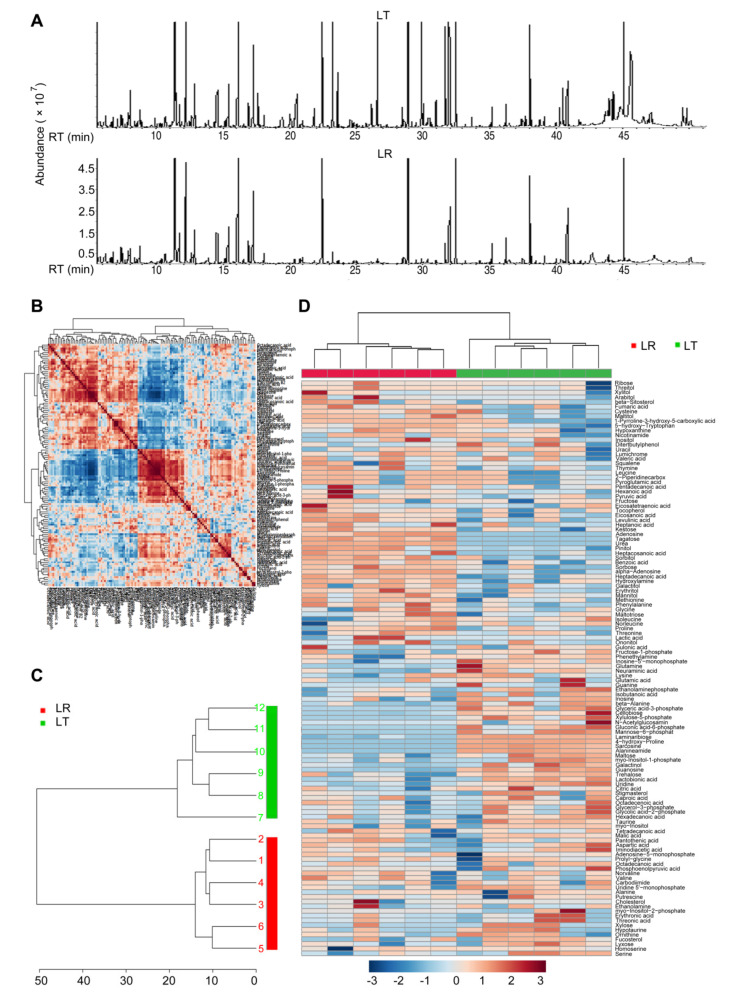
Experimental design outline and metabolomic profiling of 35 d embryos of Lantang (LT) and Landrace (LR) pigs. (**A**) Three representative total ion current chromatograms from Lantang (LT) and Landrace (LR), respectively. RT, retention time. (**B**) Reproducibility of metabolomic profiling platform used in the discovery phase. Metabolite abundances quantified in the samples over two technical replicates are shown. The correlation coefficient between technical replicates approximated to 1 (0.99905 to 0.99998). (**C**) Hierarchical cluster analysis of 12 samples. (**D**) Heat map showing the metabolites detected. Light blue and red indicate an increase or decrease in metabolites relative to the median metabolite level, respectively (see color scale).

To characterize the metabolic profiling of skeletal muscle in early embryos of local Chinese pigs and foreign lean pigs, we applied an untargeted metabolomic analysis to determine the metabolites in the LDM of LT and LR at E35. Representative total ion current chromatograms from the LT and LR samples are listed in Figure 1A. After removing internal standard ribitol and any known false positive peaks, the same compound was integrated, and the correlation coefficients of the two technique repeats indicated the accuracy of the results (Figure 1B). Using hierarchical cluster analysis, LT and LR were completely separated (Figure 1C). Metabolite profiles of the LT and LR were displayed as a heat map and 129 metabolites with reliable signals were detected in each sample (Figure 1D). Obviously, there were significant differences in the metabolite expression levels between the LT group and the LR group. For example, the levels of glutamine, laminaribiose, and gluconic acid-6-phosphate were higher in the LT group, while the levels of urea, adenosine, and mannitol were higher in the LR group (Figure 1D).

### 3.2. Differential Metabolomes Responsible for LT and LR LL

**Figure 2:**
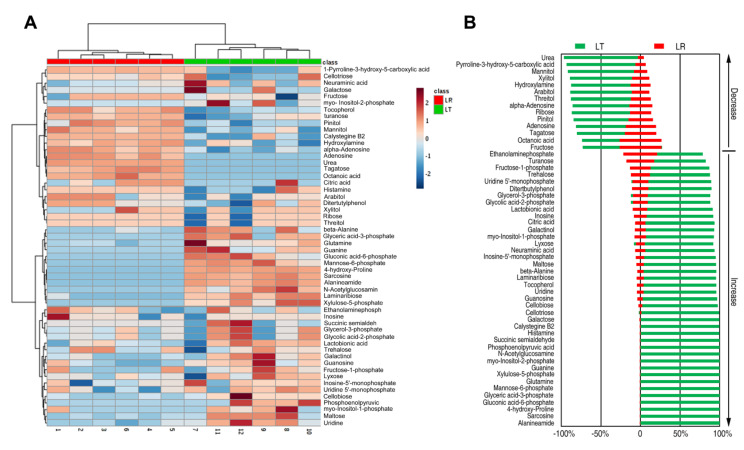
Varied metabolomes in 35 d embryos between LT and LR. (**A**) Heat map showing the relative abundance of significantly varied metabolites in LT and LR as indicated, respectively. (**B**) *Z*-values (standard deviation from average) of the LT group correspond to data in significant metabolites. *Z*-value based on the LR was calculated to identify the differential abundance of metabolites of LT compared to LR.

Using the Mann–Whitney U test, we found 54 differential metabolites (*p* < 0.05). The identified metabolites are shown in Figure 2A as a heat map. Out of the differential metabolites, 40 increased and 14 decreased for LT, and LR was the opposite (Figure 2B). These results suggested that the metabolic differences between LT and LR might be one of the reasons for the differences in embryonic skeletal muscle development between the two breeds.

### 3.3. Differentially Enriched Pathways Responsible for LT and LR LL

**Figure 3:**
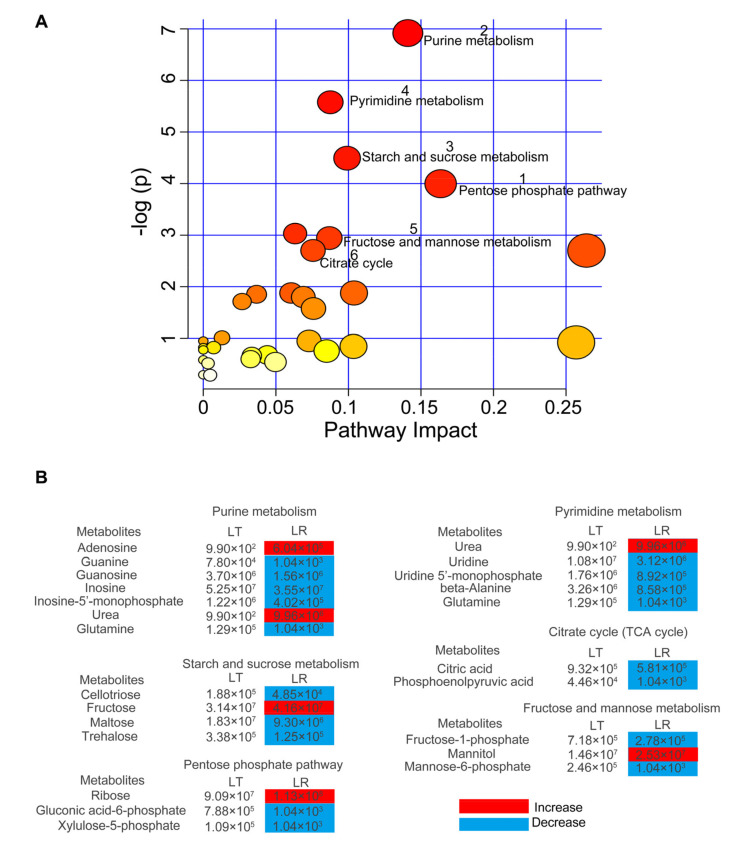
Pathway enrichment and analysis. (**A**) Pathway enrichment of various metabolites. A hypergeometric test was used to calculate the −log(*p*) value for reflecting the impact of each metabolic pathway, and pathways with *p* < 0.05 were retained. Significantly enriched pathways were selected for plotting. According to the impact value, 1 to 6 are, respectively, the pathways of pentose phosphate, purine metabolism, starch and sucrose metabolism, pyrimidine metabolism, fructose and mannose metabolism, and the citrate cycle (TCA cycle). (**B**) Integrative analysis of metabolites in significantly enriched pathways. Compared with LT, red and blue indicate an increase or decrease of metabolites in LR, respectively. Number shows the relative area of varied metabolites.

To further understand metabolome alterations, we investigated which pathways were enriched in the differential metabolites between the LT and LR groups. Using a bubble map, this investigation showed that six main pathways were enriched, including the pentose phosphate pathway, purine metabolism, starch and sucrose metabolism, pyrimidine metabolism, fructose and mannose metabolism, and the citrate cycle (TCA cycle) (Figure 3A). Importantly, differential changes of metabolites in these pathways were found. These six pathways included 22 important metabolites, among which the abundance of five metabolites (adenosine, urea, fructose, ribose, and mannitol) were increased in LR and the remaining 17 metabolites were increased in LT (Figure 3B).

### 3.4. Crucial Metabolites Responsible for LT and LR at E35

**Figure 4:**
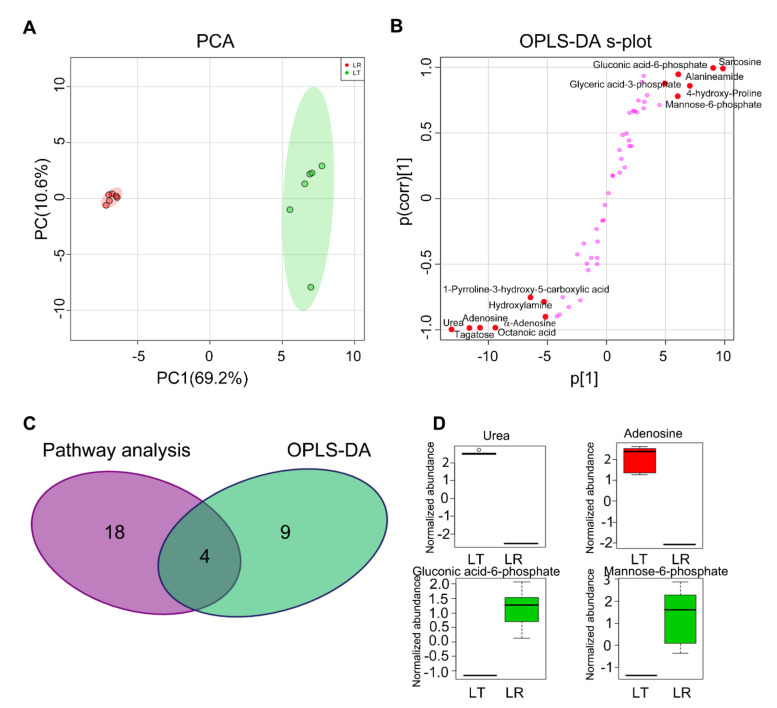
Identification of crucial metabolites. (**A**) PCA analysis of LT and LR groups according to the treatments set. Each dot represents a technological replicate analysis of samples in the plot. PC1 and PC2 used in this plot explain 79.8% of the total variance, which allows confident interpretation of the variation. PC1, principal component 1; PC2, principal component 2. (**B**) S-plot generated from OPLS-DA (R2X = 0.454, R2Y = 0.975, Q2 = 0.949). Predictive component p [1] and correlation p (corr) [1] differentiate LT from LR. Dots represent metabolites, and candidate biomarkers are highlighted in red. (**C**) Venn diagram analysis of key metabolites both in pathway enrichment analysis and OPLS-DA. (**D**) Box plot of urea, adenosine, gluconic acid-6-phosphate, and mannose-6-phosphate.

To explore the most crucial metabolites differentiating LT from LR, orthogonal partial least square discriminant analysis (OPLS-DA) was conducted to recognize the sample pattern. Results showed that LT and LR groups were distributed in two separate quarters (R2X = 0.454, R2Y = 0.975, Q2 = 0.949) by Component p [1] (Figure 4A). Discriminating variables were shown with an S-plot (Figure 4B), when the thresholds were set as the absolute values of covariance and correlation coefficients were greater than or equal to 0.05 and 0.5, respectively. Through OPLS-DA analysis, biomarkers screened by component p [1] and p (corr) [1] are shown in Figure 4B and marked in red. In addition, as demonstrated by the Venn diagram, there were four identical biomarkers in both the pathway enrichment analysis and OPLS-DA analysis (Figure 4C). Out of the four crucial metabolites, urea and adenosine were elevated in the LT group, and they had the lowest z-score. Gluconic acid-6-phosphate and mannose-6-phosphate were decreased in the LT group and had the highest z-score (Figure 4D). We speculate that these four compounds may be key metabolites for further study of embryonic muscle development.

### 3.5. Comparative Metabolic Pathway Analysis between LT and LR

**Figure 5:**
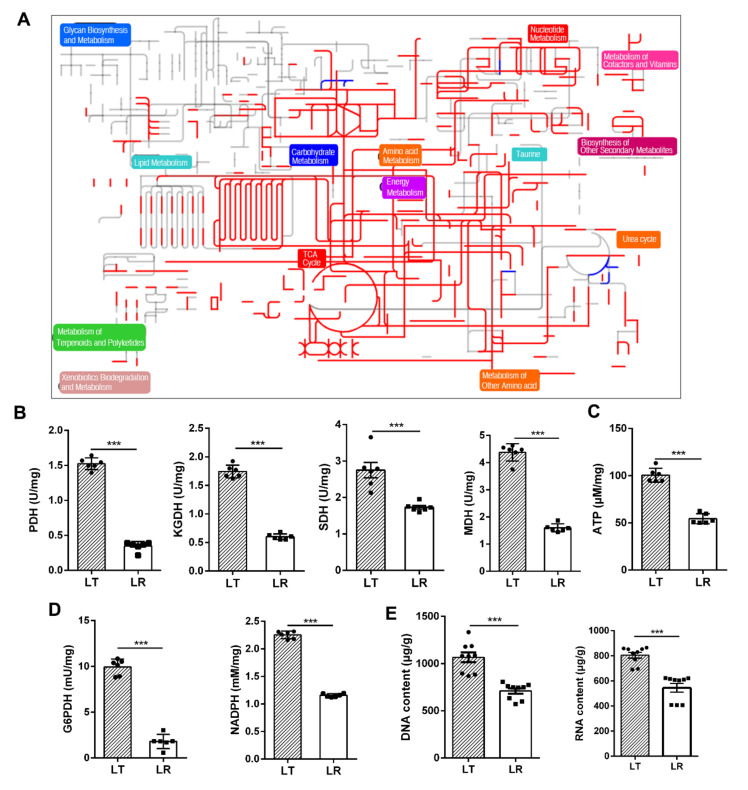
Comparative metabolic pathway analysis between LT and LR. (**A**) Analysis of the porcine metabolic profiles provides a better insight into the effects of 54 significant metabolites (*p* < 0.01) and enzyme activity. Based on the KEGG compound (http://www.kegg.jp/kegg/compound/) (accessed on 6 April 2021), metabolic network pathways are further analyzed with iPath2.0 (http://pathways.embl.de/iPath2.cgi) (accessed on 6 April 2021). Red line represents an increase in LT groups; blue line represents an increase in LT groups. (**B**) The activity of KGDH, PDH, MDH, and SDH in *LL* tissues of LT and LR. (**C**) ATP measurement in *LL* tissues of LT and LR. (**D**) The activity of G6PDH and the measurement of NADPH. (**E**) The content of DNA and RNA per unit mass of *LL* tissue metabolites in both pathway enrichment analysis and OPLS-DA. Asterisks (*) indicate levels of statistical significance. *** *p* < 0.001.

Comparative metabolic pathway analysis between the LT group and the LR group was carried out in iPath. The resulting global overview map provided a better insight into the different metabolic states, where the red line represent increased pathways in LT and the blue line represent increased pathways in LR. We identified that most of the presented pathways were elevated in LT, especially nucleotide metabolism and energy metabolism (Figure 5A). To validate the contributing role of the TCA cycle in LT muscle development, the activity of four key enzymes in the central carbon metabolism of LT and LR *LL* tissues was measured, including PDH, KGDH, SDH, and MDH. Activity of the four enzymes were elevated in LT compared with LR (*p* < 0.005, Figure 5B). In line with this, the content of ATP in LT *LL* was higher than for LR (*p* < 0.005, Figure 5C). Further, the increased activity of the G6PDH enzyme and the increased content of NADPH indicated that the pentose phosphate pathway was enhanced in LT (*p* < 0.005, Figure 5D). As shown in Figure 5E (*p* < 0.005), the content of DNA and RNA in unit weight *LL* of LT was higher than that of LR, which further proved that LT had boosted nucleotide metabolism.

## 4. Discussion

Increasing evidence shows that there is a genetic difference in the regulation of muscle development, which leads to distinct early embryonic porcine myogenesis processes between pig breeds [8,9]. Here, GC–MS-based metabolomics was used to analyze metabolic profiles in LT and LR skeletal muscle at E35. Multivariate statistical analysis of skeletal muscle metabolites showed that LT displayed a significant increase in energy metabolism and nucleotide synthesis, which might be related to the rapid myogenic differentiation of LT in the embryonic stage [22,43]. As a result, strong cell proliferation and differentiation ability requiring high energy consumption could take place.

The importance of nutrient utilization and metabolism in the process of muscle cell differentiation has been well described [44,45]. Postnatal growth rate and skeletal muscle fiber type composition of pigs are influenced by metabolic status [46,47]. It has been shown, in vitro, that different metabolic environments seriously affect the proliferation and differentiation of myoblasts [48,49]. In the current study, significant metabolomic differences were found between the *LL* tissues of LT and LR. Among the differential metabolites, in terms of purine metabolism, the increase in guanine, guanosine, and glutamine reflected a higher accumulation for purine nucleotides synthesis in LT. Likewise, in terms of pyrimidine metabolism, a higher abundance of uridine, uridine-5′-monophosphate, beta-alanine, and glutamine was found in LT. Purine metabolites and pyrimidine metabolites, serving as building blocks for DNA and RNA, are integral to the promotion of cell survival and proliferation [50]. Consistent with this, DNA and RNA content in *LL* were higher in LT pigs.

Glutamine can be used as a substrate for nucleotide synthesis (purines, pyrimidines, and amino sugars), as well as NADPH and many other biosynthetic pathways involved in the maintenance of cellular integrity and function [51]. Therefore, we speculated that the increase in glutamine might promote nucleic acid synthesis. It has previously been reported that intracellular L-glutamine levels per se play a role in the control of protein content in skeletal muscle myotube, and L-glutamine supplementation improves skeletal muscle cell differentiation [48,52]. Previous studies also report that LT show more intense myogenesis than LR at E35 [22]. Thus, the greater purine metabolism and pyrimidine metabolism could satisfy the large demand for nucleic acid substances for muscle development.

The pentose phosphate pathway (PPP) which produces ribose-5-phosphate for de novo nucleotide biosynthesis also generates NADPH, which is crucial for supporting cell proliferation via the reductive biosynthesis of macromolecules such as lipids [53,54]. In addition, NADPH functions as a reactive oxygen species (ROS) scavenger and plays an important role in cellular antioxidant defense [55]. Therefore, elevated NADPH levels contribute to the rapid proliferation and differentiation of myoblasts and may be a feature of rapid biosynthesis in LT *LL*, which contributed to the rapid proliferation and differentiation of myogenic cells. Glucose-6-phosphate dehydrogenase (G6PDH) is a rate-limiting enzyme in the PPP; it catalyzes the dehydrogenation of 6-phosphoglucose to form 6-phosphogluconate-δ-lactone [56]. The increased activity of G6PDH further demonstrated that the PPP was enhanced in LT.

The TCA cycle plays a central role in metabolism by completely oxidizing acetyl-CoA, a key product of carbohydrate, fatty acid, and amino acid catabolism, into carbon dioxide, producing ATP and meeting most of the cell’s energy requirements [57]. Metabolome analysis revealed that TCA cycle metabolites such as citric acid were increased in LT. More interestingly, we showed that ATP levels were significantly lower in LR muscle tissue. The TCA cycle consists of a series of biochemical reactions occurring in the mitochondrial matrix, in which some metabolic enzymes are involved [58,59,60]. To further demonstrate that it was boosted and the TCA cycle provided sufficient ATP for intense cell development in LT, the activity of four enzymes involved in the TCA cycle were measured. These enzymes included PDH that transforms pyruvate into acetyl-CoA, KGDH that converts α-ketoglutarate to succinyl-CoA, MDH that catalyzes the reversible conversion between oxaloacetic acid and malate, and SDH that catalyzes the transformation of succinate into fumarate [57]. According to expectations, the activity of these enzymes in LT *LL* tissue was significantly greater than in LR. These findings revealed that enhanced TCA cycles lead to the rapid generation of ATP, which could meet the energy requirements of rapid cell proliferation and differentiation in LT.

The above shows that different myogenic abilities of LT and LR pigs in early embryogenesis are related to cellular metabolic status. We found that the metabolic profiles of embryonic skeletal muscle of LT was more conducive to the rapid formation of myofiber. Although metabolic regulation of skeletal muscle development has been widely reported, it has not been further verified whether the differences in metabolic profiles between LT and LR at E35 are the cause or consequence of the differences in skeletal muscle development. It is also unclear whether the differences affect muscle mass at a later stage. This is the deficiency of this study. In addition, the mechanism underlying the metabolome differences is unclear; this may be owing to several factors. As the alterations were observed in embryos whose parents were raised under uniform conditions, different levels of metabolism may be linked with a genetic or epigenetic origin. For example, the occurrence of severe obesity is related to differing methylation modification signatures near the gene promoter of several metabolic enzymes, resulting in altered gene expression [61,62]. It has also been hypothesized that post-translational modification (i.e., acetylation and phosphorylation) of metabolic enzymes can impair TCA cycle flux or cause metabolic disorders [63,64]. A limitation of the current study was not directly examining either of these factors. Although we revealed that the activity of several metabolic enzymes involved in the TCA cycle and PPP in LT pigs was significantly stronger than that in LR pigs, the underlying mechanism remains unclear. In addition, the TCA cycle occurs in cell mitochondria, so the increased or decreased content of mitochondria in skeletal muscle cells may contribute to the changed TCA cycle flux [65,66].

## 5. Conclusions

In conclusion, GC–MS-based metabolomics was adopted to understand the mechanisms by which LT had a more intense myogenic capacity than LR at the early embryonic stage. Results showed that abudances of individual metabolites were clearly different in the *LL* tissues of LT and LR. Furthermore, comparative metabolic pathway analysis and enzyme activity detection revealed that nucleotide metabolism and energy metabolism, including purine metabolism, pyrimidine metabolism, PPP, and the TCA cycle, were increased in LT, permitting rapid generation of nucleic acids and ATP, to meet the material and energy requirements of rapid cell proliferation and differentiation. By OPLS-DA and pathway enrichment analysis, four crucial biomarkers were identified that may have important effects on porcine muscle development. To our knowledge, this is the first report that reveals the relationship between functional metabolomics and differences in embryonic muscle development among pig breeds. It will provide a new insight for elucidating the regulatory mechanism concerning porcine embryonic muscle development.

## Data Availability

The original data can be obtained by contacting the corresponding author.
